# The Efficacy of Lubiprostone in Patients of Constipation: An Updated Systematic Review and Meta‐Analysis

**DOI:** 10.1002/jgh3.70070

**Published:** 2025-01-15

**Authors:** Umar Akram, Obaid Ur Rehman, Eeshal Fatima, Zain Ali Nadeem, Omer Usman, Waqas Rasheed, Ramsha Ali, Khawaja Abdul Rehman, Abdulqadir J. Nashwan

**Affiliations:** ^1^ Department of Medicine Allama Iqbal Medical College Lahore Pakistan; ^2^ Department of Medicine Services Institute of Medical Sciences Lahore Pakistan; ^3^ Department of Internal Medicine Texas Tech University Health Sciences Center El Paso/Transmountain Texas USA; ^4^ Department of Medicine University of Kentucky Lexington Kentucky USA; ^5^ Department of Medicine People's University of Medical and Health Sciences Nwabshah Shaheed Benazirabad Pakistan; ^6^ Department of Medicine CMH Lahore Medical College and Institute of Dentistry Lahore Pakistan; ^7^ Department of Nursing Hamad Medical Corporation Doha Qatar

**Keywords:** chronic idiopathic constipation, constipation, irritable bowel syndrome, lubiprostone, opioid induced constipation

## Abstract

**Background and Aim:**

Lubiprostone increases chloride and water secretion in the intestines, and several studies have demonstrated the efficacy of lubiprostone in treating functional constipation. Several new clinical trials have emerged since the previous meta‐analysis conducted in 2020. We conducted this updated meta‐analysis to assess clinical efficacy of lubiprostone in these patients.

**Methods:**

A systematic search was conducted on MEDLINE, Cochrane, and Scopus. Randomized controlled trials published between July 2019 and June 2024 were selected. Cochrane's RoB 2 tool was used to assess the risk of bias. A meta‐analysis was performed and findings were presented using forest plots.

**Results:**

A total of 14 studies, comprising 4550 patients, were included in the review. Only 12 studies were pooled in the meta‐analysis. Lubiprostone was associated with greater spontaneous bowel movements (SBM) per week (RR 1.454, 95% CI 1.193–1.771) and SBM within 24 h (RR 1.790, 95% CI 1.491–2.150) in patients with chronic idiopathic constipation (CIC). However, it was not associated with abdominal pain in either arm (RR 1.415, 95% CI 0.873–2.294). In opioid‐induced constipation (OIC), lubiprostone increased SBM within 24 h (RR 1.277, 95% CI 1.105–1.475) but did not significantly affect abdominal pain (RR 4.321, 95% CI 0.624–29.941). Lubiprostone improved all selected SBM‐related and abdominal pain outcomes in patients with irritable bowel syndrome with constipation (IBS‐C).

**Conclusion:**

Lubiprostone significantly improves all SBM‐related outcomes. Owing to its good safety and efficacy profile, lubiprostone can be used in the combination regimens for management of CIC, IBS‐C, and OIC.

## 1 Introduction

Constipation is a common issue affecting individuals of all ages, including children and adults. In the United Kingom, around one in seven adults and one in three children experience constipation at any given time [1]. Constipation also impacts the economy heavily—the United States spends over 800 million dollars annually on laxatives [2]. In addition to its impact on quality of life, constipation reduces an individual's productivity and increases the frequency of missing work or school days [3]. Chronic idiopathic constipation (CIC) is a common functional disorder impacting up to 17% of the global population [4], with a higher prevalence among females and older individuals [5]. Most CIC patients rely on traditional laxatives for relief; the usage of laxatives is linked to age, the frequency of symptoms, and the duration of constipation [6]. Constipation is also a substantial concern in individuals with irritable bowel syndrome (IBS) [7]. Prominent symptoms of irritable bowel syndrome with constipation (IBS‐C) include abdominal pain, bloating, and straining [8]. Opioid‐induced constipation (OIC) is a frequent problem encountered by patients on long‐term opioid therapy, affecting their quality of life [9]. OIC is mainly associated with cancer pain, chronic non‐cancer pain, and opioid dependence treatment [10].

Constipation may result from mechanical obstruction of the gastrointestinal tract, a low‐fiber diet, lack of exercise, neuromuscular disorders, metabolic abnormalities, or the use of certain medications such as opiate analgesics, anticholinergic antidepressants, and calcium channel blockers [11]. Regardless of the underlying cause, the main goals of treatment are to reduce patient discomfort and return bowel function to normal [12]. For patients with functional constipation, a variety of laxatives are available, including prokinetics and osmotic, stimulant, or secretory laxatives [13]. However, existing treatment options do not adequately control IBS‐C symptoms due to high treatment costs and increased utilization of healthcare resources [14]. A novel, efficacious, and well‐tolerated treatment is necessary for constipation in patients diagnosed with CIC, IBS‐C, or OIC [15].

Lubiprostone, an emerging treatment option for functional constipation, works by increasing the release of chloride and water in the intestines [16]. It is categorized as a secretory laxative and belongs to the novel class of compounds referred to as prostones [17]. Previous investigations in healthy volunteers and constipation patients have demonstrated lubiprostone's efficacy in increasing bowel movement frequency and alleviating other constipation symptoms [18]. Several studies have investigated the efficacy of lubiprostone for the treatment of constipation; a meta‐analysis conducted in 2020 revealed positive outcomes [19]. However, results from several new, well‐designed clinical trials have been published [20, 21, 22]. We conducted this updated meta‐analysis to better analyze the efficacy and safety of Lubiprostone in CIC, IBS‐C, and OIC patients.

## 2 Methods

The guidelines of the Cochrane Handbook for Systematic Reviews of Interventions were used to conduct this systematic review, and reported according to the Preferred Reporting Items for Systematic Reviews and Meta‐analysis (PRISMA) statement [23]. This review has been registered with the International Prospective Register of Systematic Reviews (PROSPERO) under the identifier CRD42023443405. Our study did not require ethical approval.

### 2.1 Data Sources and Search Strategy

Two reviewers independently searched for eligible studies on the electronic databases and clinical trial registers: MEDLINE (PubMed), Cochrane Central Register of Controlled Trials (CENTRAL), Google Scholar, and ClinicalTrials.gov from July 2019 to June 2024. The primary keywords included “lubiprostone,” “constipation,” “chronic idiopathic constipation,” “opioid‐induced constipation,” and “irritable bowel syndrome.” Variations of the keywords were combined with the appropriate Boolean operators. A detailed overview of the search strategy is shown in Table S2. No language restriction was applied. We searched for additional articles by screening the reference lists of obtained studies and similar clinical trials.

### 2.2 Eligibility Criteria

We included the studies if they: (i) were randomized controlled trials (RCTs), (ii) involved patients diagnosed with CIC, OIC, or IBS‐C, (iii) reported spontaneous bowel movements and abdominal pain or discomfort as outcomes, and (iv) were available online in English. Studies were excluded due to the following reasons: (i) study designs other than randomized controlled trials, (ii) not comparing the efficacy of lubiprostone with a control group, (iii) not available in English. Additionally, we excluded editorials, reviews, conference abstracts, and experimental studies on animal models. The definitions of CIC, OIC, and IBS‐C were adopted from the original studies, provided that it was clearly mentioned in the available manuscript.

### 2.3 Selection Process

Mendeley Desktop 1.19.8 (Mendeley Ltd., Amsterdam, The Netherlands) was employed for the deduplication and screening of all the articles retrieved through our online search. After deduplication, two authors independently completed the first phase of screening titles and abstracts. The remaining articles were then subjected to comprehensive full‐text screening by the same authors. Any disagreements between them were resolved by a third reviewer.

### 2.4 Outcomes Measured

The following outcomes were measured to assess the efficacy of lubiprostone: (i) frequency of spontaneous bowel movements (SBM), assessed as mean change from baseline, frequency, and number of patients having an SBM within 24 h of the first dose of lubiprostone; (ii) full‐responder rate, defined as the number of patients presenting > 3–4 SBMs in a week; and (iii) degree of abdominal pain or discomfort, assessed as mean score and number of patients experiencing the symptoms.

SBM was defined as spontaneous bowel movement occurring 24 h or more after the use of rescue medication. Abdominal pain or discomfort was rated using a Likert scale with 0 indicating “absent,” 1 indicating “mild,” 2 indicating “moderate,” 3 indicating “severe,” and 4 indicating “very severe.” Abdominal pain as an outcome was assessed in studies having a patient population diagnosed with IBS‐C whereas abdominal discomfort was assessed in CIC and OIC trials due to the different clinical manifestations of these conditions.

### 2.5 Data Extraction and Quality Assessment

A spreadsheet was designed to extract relevant data from the included studies including the primary outcomes defined above, and other study characteristics such as the first author's name, year, sample size, and the disease (CIC, OIC, IBS‐C). The risk of bias was assessed using Cochrane's RoB 2, a revised tool for assessing the risk of bias in RCTs. RoB 2 uses five domains to determine the overall risk of bias: bias due to problems in the randomization process, bias due to deviations from the intended outcomes, bias due to missing outcome data, bias due to problems in the measurement of data, and bias due to selective reporting of the results. Two authors independently rated the risk of bias for each included study as low, high, or some concerns. Any disagreement between them was resolved by a third reviewer.

### 2.6 Data Analysis

Comprehensive Meta‐Analysis software, version 3.3 (Biostat Inc., Englewood, NJ, USA) was used for the statistical analysis. We calculated risk ratios (RR) and their 95% confidence intervals (95% CI) for dichotomous events using the Mantel–Haenszel method and a random effects model. Those studies not providing sufficient information for pooling results for continuous outcomes, such as median time to first SBM and abdominal pain and discomfort scales, were not included in the statistical analysis. Between‐study heterogeneity was assessed by Cochran's Q test and the I2 statistic; a *p*‐value < 0.05 was considered statistically significant. Doi plots and Luis Furuya‐Kanamori (LFK) index were used to assess publication bias because it has greater sensitivity and power than Egger's test for outcomes < 10 studies [24].

## 3 Results

### 3.1 Study Selection

**FIGURE 1:**
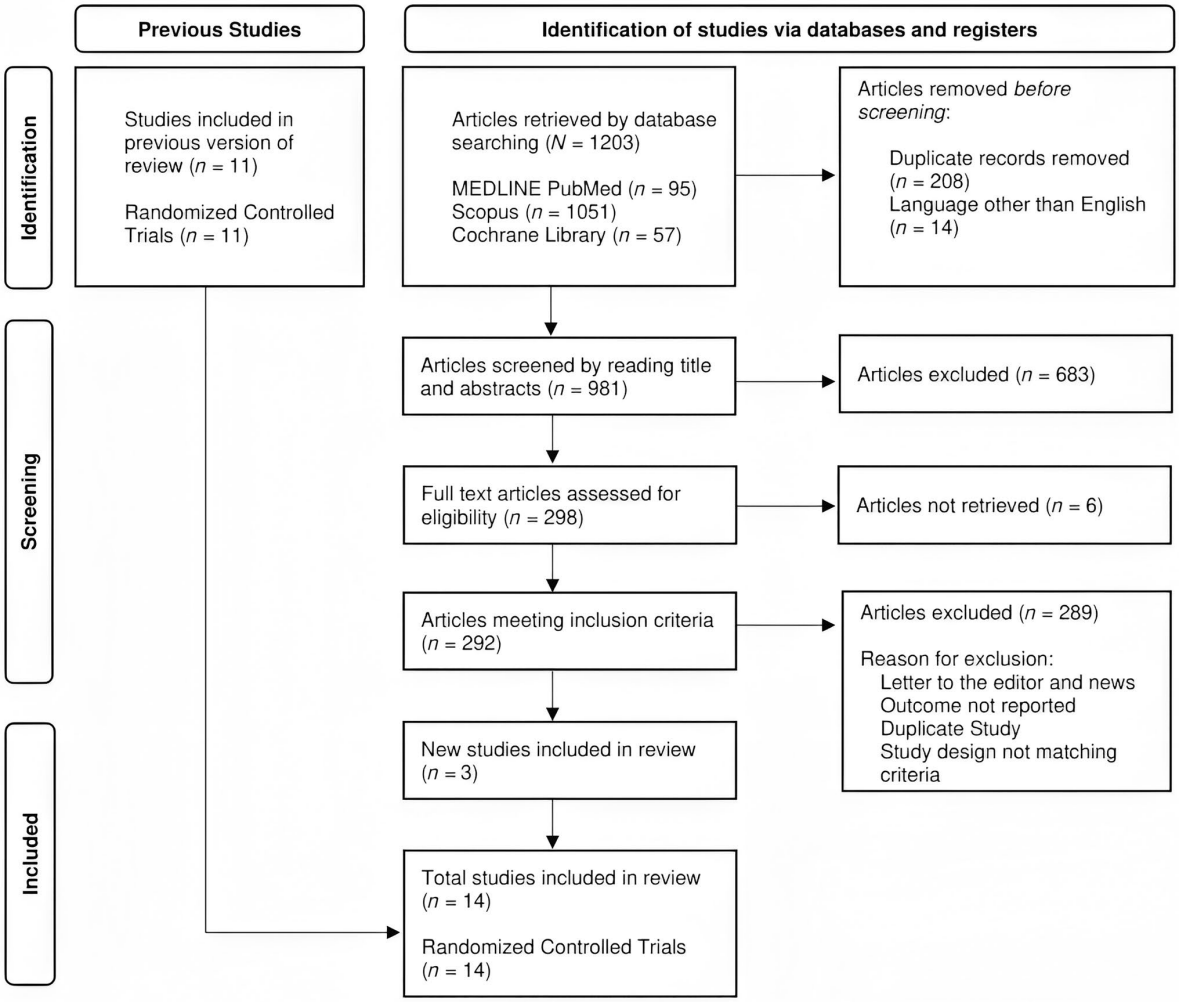
PRISMA flow diagram.

We retrieved the 11 studies included in the previous meta‐analysis and searched the databases for articles published after July 2019. A total of 1203 articles were initially retrieved. After removing duplicates, the title and abstracts of 981 articles were screened, and 298 articles were selected for a full‐text review. We found three new RCTs which were relevant. In total, 14 studies met the inclusion criteria and are included in this version of the review. Figure 1 shows the PRISMA flow chart summarizing the study selection process. The studies comprised a total of 4550 patients—involving 1884 CIC patients, 1366 IBS‐C patients, and 1300 OIC patients. Of the 14 studies, 12 studies were included in the meta‐analysis. The earliest study was published in 2007 and the latest study was published in 2022.

### 3.2 Quality of the Included Studies

**FIGURE 2:**
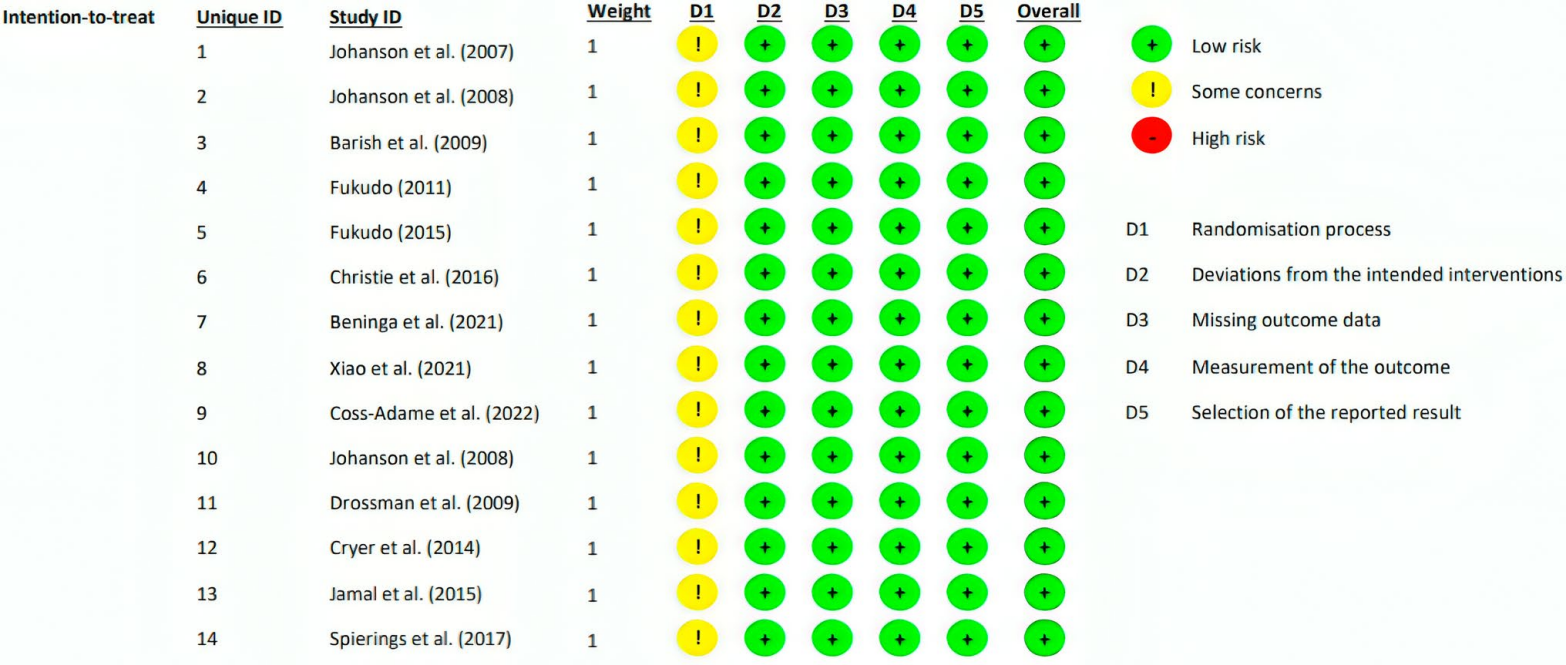
Quality assessment of the included studies.

Figure 2 shows the result of the quality assessment. While there were some concerns in the randomization process of all studies, the overall risk of bias was low.

### 3.3 Outcomes in CIC Studies

**FIGURE 3:**
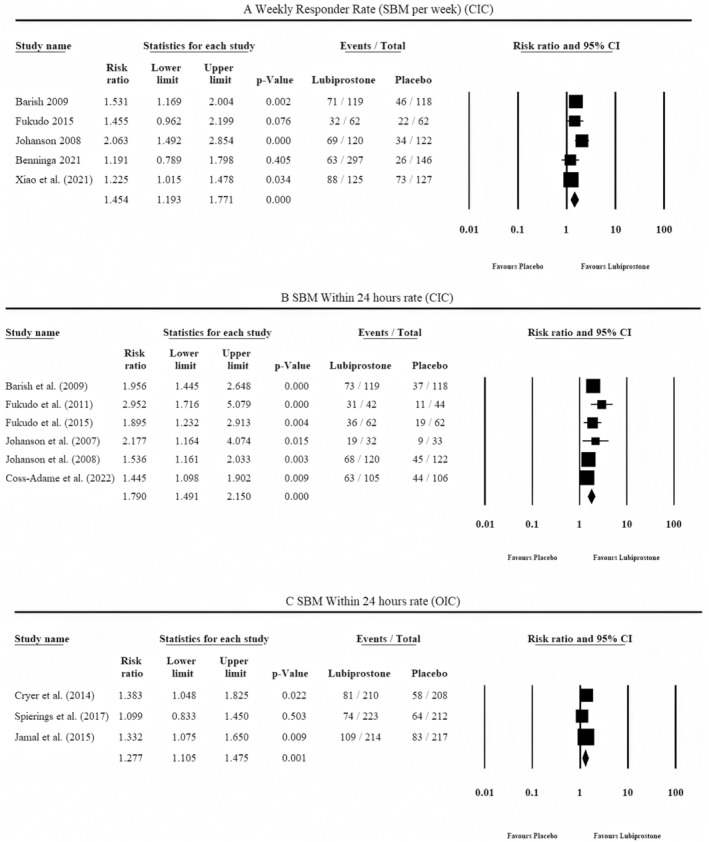
Outcome analysis in the included studies.

Among CIC studies (Table 1), five studies reported data for full‐responder rate. According to the pooled results shown in Figure 3A, lubiprostone was associated with a significantly greater full‐responder rate (RR = 1.454, 95% CI = 1.193–1.771, *p* = 0.000, I2 = 53%). No asymmetry was observed in the doi plot (LFK index = 0.17), suggesting no evidence of publication bias (Figure S1). Six studies reported data for SBM within 24 h. According to the pooled results shown in Figure 3B, lubiprostone significantly increased the frequency of SBM within 24 h (RR = 1.790, 95% CI = 1.491–2.150, *p* = 0.000, I2 = 32%). Minor asymmetry was observed in the doi plot (LFK index = 1.39), suggesting little to no evidence of publication bias (Figure S2).

**TABLE 1:** Main findings in CIC lubiprostone studies.

	Outcomes	Studies	Results (lubiprostone vs. placebo)	*p*
**CIC**	Mean change in the number of SBMs from baseline (4 weeks)	Fukudo, 2015	2.56 versus 1.62	0.042
		Xiao, 2021	3.14 versus 1.92	0.0006
	Mean SBM per week (8 weeks)	Christie, 2016	7.00 versus 5.27	0.02
	Mean SBM per week (4 weeks)	Johanson, 2008	5.30 versus 2.91	0.002
		Coss‐Adame, 2022	7.1 versus 5.6	0.013
		Xiao, 2021	4.46 versus 3.24	0.0006
		Barish, 2009	5.37 versus 3.46	0.0068
		Christie, 2016	5.77 versus 4.78	NR
	Mean SBM per week (1 week)	Johanson, 2007	Point estimates NR, higher mean for lubiprostone (chart)	0.02
		Coss‐Adame, 2022	6.7 versus 5.2	NR
		Xiao, 2021	4.88 versus 3.22	< 0.0001
		Johanson, 2008	5.69 versus 3.46	0.0001
		Barish, 2009	5.89 versus 3.99	0.0001
	Change in mean SBM in the first week	Fukudo, 2011	6.8 versus 1.5	0.0001
		Fukudo, 2015	3.7 versus 1.3	0.001
		Coss‐Adame, 2022	4.9 versus 3.0	0.02
		Xiao, 2021	3.55 versus 1.90	< 0.0001
	SBM within 24 h	Johanson, 2007	59.4% versus 27.3%	0.009
		Johanson, 2008	56.7% versus 36.9%	0.0024
		Barish, 2009	61.3% versus 31.4%	0.0001
		Fukudo, 2011	75.0% versus 26.2%	0.0001
		Fukudo, 2015	58.1% versus 34.6%	0.004
		Coss‐Adame, 2022	60.0% versus 41.5%	0.009
	Weekly full‐responder rate^a^ (≥ 3–4 SBM per week) (4 weeks)	Johanson, 2008	57.8% versus 27.9%	0.004
		Xiao, 2021	62.31% versus 47.29%	0.0178
		Barish, 2009	60.0% versus 39.0%	0.0022
		Fukudo, 2015	54.2% versus 36.7%	0.066
	Mean abdominal discomfort score (4 weeks)	Johanson, 2008	1.23 versus 1.52	0.045
		Barish, 2009	1.24 versus 1.47	0.1383
		Coss‐Adame, 2022	0.9 versus 1.2	0.004
	Mean abdominal discomfort rate (4 weeks)	Christie, 2016	53.0% versus 67.0%	0.86
	Mean abdominal discomfort rate (8 weeks)	Christie, 2016	50.0% versus 52.0%	0.86
	Overall SBM response	Beninga, 2021 (study 1 mITT population)	18.5% versus 14.4%	0.2245

Abbreviations: CIC: chronic idiopathic constipation; NR: not reported; SBM: spontaneous bowel movements.

^a^ Definition of full‐responder = ≥ 3 SBM per week.

**FIGURE 4:**
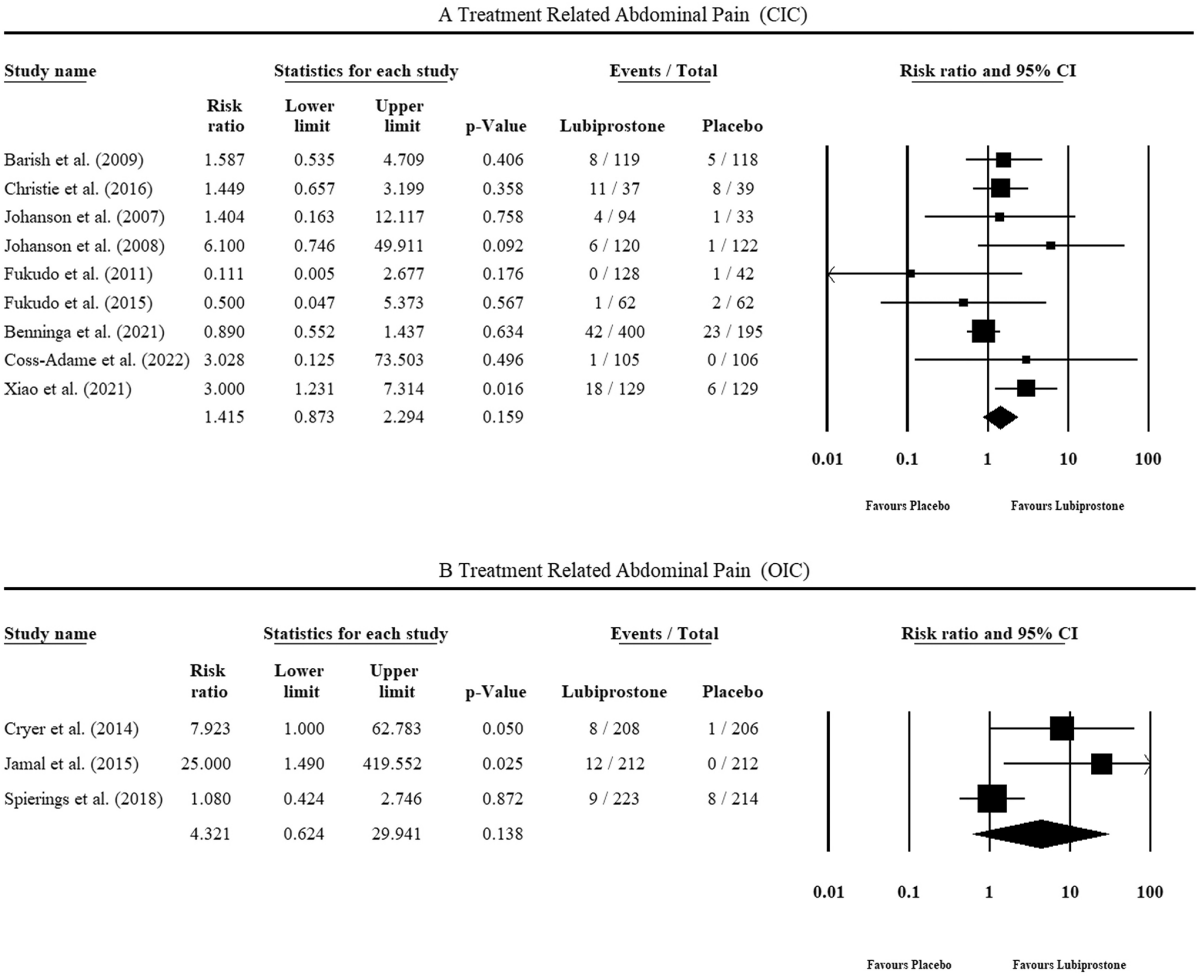
Treatment‐related abdominal pain.

All CIC studies reported adverse events during treatment. Christie et al. (2016), Fukudo et al. (2011) and Fukudo et al. (2015) (Figure 4A) found no significant association of abdominal pain with either group. Johanson et al. 2007 and 2008 reported significant gastrointestinal disorders in the lubiprostone arm, and Xiao et al. noted the statistically significant association of lubiprostone administration with abdominal pain. The pooled results showed no significant association of abdominal pain with either arm (RR = 1.415, 95% CI = 0.873–2.294, *p* = 0.159, I2 = 29%). There was low heterogeneity between studies. No asymmetry was observed in the doi plot (LFK index = 0.15), suggesting no evidence of publication bias (Figure S3).

### 3.4 Outcomes in IBS‐C Studies

In extended follow‐ups (up to 3 months) in the IBS‐C studies (Table 2), all selected SBM‐related outcomes were significantly better among lubiprostone patients. Three RCTs evaluated the mean scores for measures of abdominal pain at 1, 2, and 3 months following the start of treatment. After 2 and 3 months of taking lubiprostone, individuals in the Drossman et al. study showed more improvement in abdominal pain than those taking a placebo. After 1 and 2 months of lubiprostone medication, Johanson et al. observed a greater mean improvement in the abdominal pain score compared to the baseline.

**TABLE 2:** Main findings in IBS‐C lubiprostone studies.

	Outcomes	Studies	Results (lubiprostone vs. placebo)	*p*
**IBS‐C**	Overall responder rate^a^	Drossman, 2009	17.9% versus 10.1%	0.001
	Weekly responder rate (weeks 2, 4, 5, 6, 10, and 12)	Drossman, 2009	Point estimates NR, higher rate for lubiprostone (chart)	0.030
	Monthly responder rate (month 3)	Study 0431 in Drossman, 2009	21.3% versus 14.5%	0.026
		Study 0432 in Drossman, 2009	22.7% versus 14.6%	0.026
		Drossman, 2009 (pooled results)	22.0% versus 14.5%	0.003
	Mean change from baseline in weekly SBM rate (month 3)	Johanson, 2008	Point estimates NR, higher rate for lubiprostone (chart)	0.033
	Mean improvement in abdominal pain score (month 1)	Johanson, 2008	Point estimates NR, higher mean for lubiprostone (chart)	0.023
		Drossman, 2009	Point estimates NR, higher mean for lubiprostone	> 0.05
	Mean improvement in abdominal pain score (month 2)	Johanson, 2008	Point estimates NR, higher mean for lubiprostone (chart)	0.028
		Drossman, 2009	−0.43 versus −0.35	0.039
	Mean improvement in abdominal pain score (month 3)	Johanson, 2008	Point estimates NR, higher mean for lubiprostone (chart)	0.260
		Drossman, 2009	−0.45 versus −0.36	0.028

Abbreviations: IBS‐C: constipation‐predominant irritable bowel syndrome; NR: not reported; SBM: spontaneous bowel movements.

^a^ Responder rate was defined as patients achieving ≥ 3–4 SBM per week.

### 3.5 Outcomes in OIC Studies

Among the OIC Studies (Table 3), three trials reported data for SBM within 24 h. The pooled results in Figure 3C show that lubiprostone significantly increased the frequency of SBM within 24 h (RR = 1.277, 95% CI = 1.105–1.475, *p*‐value = 0.001, I2 = 0%). Minor asymmetry was observed in the doi plot (LFK index = −1.25), suggesting little to no evidence of publication bias (Figure S4). Additionally, lubiprostone yielded favorable results for SBM at week 8 (1 out of 2 RCTs) and week 12 (2 out of 3 RCTs), and overall responder rate (2 out of 3 RCTs). Publication bias could not be assessed as the number of included studies for this analysis was less than 10.

**TABLE 3:** Main findings in OIC lubiprostone studies.

	Outcomes	Studies	Results (lubiprostone vs placebo)	*p*
**OIC**	Mean change from baseline in SBM frequency (week 12)	Cryer, 2014	Point estimates NR, higher mean for lubiprostone (chart)	0.091
		Jamal, 2015	Point estimates NR, higher mean for lubiprostone (chart)	0.040
		Spierings, 2017	2.5 versus 2.6	0.956
	Mean change from baseline in SBM frequency (week 8)	Cryer, 2014	3.3 versus 2.4	0.005
		Spierings, 2017	2.6 versus 2.4	0.842
	Mean change from baseline in SBM frequency (overall)	Cryer, 2014	2.2 versus 1.6	0.004
		Jamal, 2015	3.2 versus 2.4	0.001
		Spierings, 2017	2.6 versus 2.3	0.224
	Overall responder rate	Jamal, 2015	27.1% versus 18.9%	0.030
	SBM within 24 h	Cryer, 2014	38.8% versus 27.8%	0.018
		Jamal, 2015	50.9% versus 38.2%	0.008
		Spierings, 2017	33.2% versus 30.2%	0.502
	Mean improvement in abdominal discomfort scales (overall)	Cryer, 2014	Point estimates NR, higher mean for lubiprostone (chart)	0.047
		Jamal, 2015	Point estimates NR, higher mean for lubiprostone (chart)	0.127
		Spierings, 2017	−0.5 versus −0.4	0.027
	Median time to first SBM	Cryer, 2014	28.5 versus 46.0 h	0.053
		Jamal, 2015	23.5 versus 37.7 h	0.004

Abbreviations: NR: not reported; OIC: opioid‐induced constipation; SBM: spontaneous bowel movement.

All OIC studies also reported treatment‐associated adverse events. Cryer et al. observed a statistically significant association between the incidence of abdominal pain and lubiprostone administration (Figure 4B). Jamal et al. reported significantly higher gastrointestinal disorders in the lubiprostone arm, and the placebo group had no incidence of abdominal pain. Spierings et al. noted no significant association of gastrointestinal disorders with either group. A pooled analysis proved no significant association of abdominal pain with either group (RR = 4.321, 95% CI = 0.624–29.941, *p*‐value = 0.138, I2 = 69%). A moderate heterogeneity was observed between studies. Major asymmetry was observed in the doi plot (LFK index = 4.67), suggesting evidence of publication bias (Figure S5).

## 4 Discussion

We conducted an updated systematic review and meta‐analysis of randomized controlled trials [17, 20, 21, 22, 25, 26, 27, 28, 29, 30, 31, 32, 33, 34] to assess the efficacy of Lubiprostone in patients of CIC, IBS‐C, and OIC. The weekly responder rate was reported by five studies [17, 20, 22, 26, 30] and the meta‐analysis favored lubiprostone over placebo among the CIC patients. Six studies [17, 21, 26, 27, 30, 31] reported SBM within 24 h among CIC patients, and the meta‐analysis favored lubiprostone over placebo. In the OIC studies [25, 29, 33], SBM was reported within 24 h, and the combined effect estimate favored lubiprostone. Among the IBS‐C studies [28, 32], a trend favoring lubiprostone was observed. Treatment‐related abdominal pain was reported by CIC studies and OIC studies; however, pooled analysis revealed that lubiprostone was not significantly associated with increased risk of abdominal pain. There was moderate heterogeneity in the CIC studies for the primary outcome which could be attributed to the differences in baseline statistics of the patients such as age, race, ethnicity, and comorbidities. For example, the mean age varied significantly across studies, ranging from 10.67 years in Benninga et al. [22] to 48.56 years in Johanson et al. [26], while the male‐to‐female ratio was also notably different, with a low proportion of males in Johanson et al. [26] (10.29%) compared to a much higher ratio in Benninga et al. [26] (84.57%).

The results of our meta‐analysis are comparable with previous literature exploring the efficacy of lubiprostone in patients of CIC, OIC, and IBS‐C. A previous meta‐analysis conducted by Passos et al. [19] demonstrated a significant improvement in weekly responder rate and SBM within 24 h rate in CIC studies, which is consistent with our findings. However, their pooled analysis did not reveal a significant association of lubiprostone with improved SBM within 24 h rate in OIC studies, which is in contrast with our findings. Furthermore, we also identified a nonsignificant association of lubiprostone with treatment‐related abdominal pain in CIC as well as OIC studies. However, the previous investigation by Passos et al. [19] did not report this outcome.

Additionally, the United States Food and Drug Administration approved lubiprostone (24 μg twice daily) for CIC after a positive trend was observed favoring lubiprostone over placebo in two randomized double‐blind placebo‐controlled clinical trials consisting of 479 patients [35]. Additionally, lubiprostone resulted in constant improvement of abdominal bloating and constipation compared to the baseline in the long term as seen in three subsequent open‐label trials comprising 871 CIC patients [35]. For IBS‐C, the approved dose of lubiprostone is 8 μg twice daily. Long‐term effects of lubiprostone use in IBS‐C have not been explored yet. Current practice is to prescribe lubiprostone for a 12‐week trial period; if the patient fails to respond to lubiprostone within this trial period, the treatment is discontinued [32]. The approval of lubiprostone was based on the results of two large multicenter, placebo‐controlled RCTs including 1154 adults, which showed a better overall response rate for 12‐week lubiprostone use. No statistically significant adverse events were observed in these trials [32]. The follow‐up open‐label trials among IBS‐C patients yielded increased efficacy of lubiprostone at 52 weeks [36]. A pooled analysis in a post hoc study [37] showed that response rates for abdominal pain and bloating are significantly higher in patients on lubiprostone as compared to placebo for IBS‐C. In the relative individual trials [28, 32] included in this review exploring the efficacy of lubiprostone in IBS‐C patients, there was a mean improvement in the symptoms of abdominal pain and bloating at about 8 weeks of treatment. For OIC, the results obtained based on individual analysis of the three included studies, Cryer et al. [33] and Jamal et al. [29] showed a positive trend in terms of overall response favoring lubiprostone over placebo. Spierings et al. [25] did not show any statistically significant improvement in responder rate associated with lubiprostone. However, the overall pooled results provided in our meta‐analysis showed a statistically significant responder rate favoring lubiprostone. Adverse effects are not common with lubiprostone use and the drug is well tolerated. Consistent with previous studies, we observed no significant relationship between abdominal pain and lubiprostone use in OIC patients [38]. Recent literature shows some other adverse effects commonly observed in patients taking lubiprostone, including headache, chest discomfort, and peripheral edema [39].

Our study has certain limitations that should be considered when interpreting the results. First, we observed some heterogeneity between the studies, possibly due to varying duration of follow‐up, different criteria for the outcomes, and discrepancies in baseline characteristics of the patients. Second, most of the included studies had short follow‐up durations. Third, we exclusively considered published articles, excluding conference abstracts and gray literature. Fourth, we limited our study to articles available in English, excluding any publications in other languages.

Although lubiprostone presents a significant success rate in improving SBM and alleviating abdominal discomfort in patients with CIC, IBS‐C, and OIC versus placebo, we suggest future studies to compare its efficacy with conventional treatment regimens. Future trials should incorporate longer follow‐up periods to better predict real‐life outcomes and adverse events. Lastly, combination regimens containing lubiprostone should be explored.

## 5 Conclusion

Lubiprostone significantly improves all SBM‐related outcomes and is effective for the management of CIC, IBS‐C, and OIC. We found no association of gastrointestinal adverse events with lubiprostone use. Lubiprostone demonstrated a good safety profile and can be used in combination regimens for the treatment of constipation.

## Ethics Statement

Ethical approval is not applicable to this study type. All authors have read and approved the final version of the manuscript.

## Conflicts of Interest

The authors declare no conflicts of interest.

## Supporting information


**Supplementary Figure S1:** Doi plot of SBM Per week (CIC).
**Supplementary Figure S2:** Doi plot of SBM within 24 h (CIC).
**Supplementary Figure S3:** Doi plot of Treatment Related Abdominal Pain (CIC).
**Supplementary Figure S4:** Doi plot of SBM within 24 h (OIC).
**Supplementary Figure S5:** Doi plot of Treatment Related Abdominal Pain (OIC).
**Supplementary Table S1:** Detailed Search Strategy of Each Database.

## Data Availability

The data are available upon reasonable request from the corresponding author.
