# Supplementary material for: The Efficacy of Lubiprostone in Patients of Constipation: An Updated Systematic Review and Meta‐Analysis
Source: JGH Open. 2025 Jan 15;9(1):e70070. doi: 10.1002/jgh3.70070 (PMC11735734; doi:10.1002/jgh3.70070)

**Supplementary Material**

**The Efficacy of Lubiprostone in Patients of Constipation: An Updated Systematic Review and Meta-Analysis**

This supplemental material has been provided by the authors to give readers additional information about their work.

**Supplementary Table S1:** Detailed Search Strategy of Each Database

| **Database** | **Search Strategy** | **Results** |
| --- | --- | --- |
| MEDLINE  (via PubMed) | (("lubiprostone"[MeSH Terms] OR "lubiprostone"[All Fields]) AND ("constipation"[MeSH Terms] OR "constipation"[All Fields] OR "constipated"[All Fields] OR "constipating"[All Fields] OR "constipations"[All Fields] OR (("chronic"[All Fields] OR "chronical"[All Fields] OR "chronically"[All Fields] OR "chronicities"[All Fields] OR "chronicity"[All Fields] OR "chronicization"[All Fields] OR "chronics"[All Fields]) AND ("idiopathic"[All Fields] OR "idiopathically"[All Fields] OR "idiopathics"[All Fields]) AND ("constipation"[MeSH Terms] OR "constipation"[All Fields] OR "constipated"[All Fields] OR "constipating"[All Fields] OR "constipations"[All Fields])) OR ("opioid induced constipation"[MeSH Terms] OR ("opioid induced"[All Fields] AND "constipation"[All Fields]) OR "opioid induced constipation"[All Fields] OR ("opioid"[All Fields] AND "induced"[All Fields] AND "constipation"[All Fields]) OR "opioid induced constipation"[All Fields]) OR "IBS-C"[All Fields] OR ("irritable bowel syndrome"[MeSH Terms] OR ("irritable"[All Fields] AND "bowel"[All Fields] AND "syndrome"[All Fields]) OR "irritable bowel syndrome"[All Fields]))) AND (2019:2023[pdat]) | 95 |
| Cochrane Central | Lubiprostone AND constipation | 57 Trials |
| Scopus | lubiprostone AND constipation OR "Chronic Idiopathic Constipation" OR "Opioid Induced Constipation" OR ibs-c OR "irritable bowel syndrome" | 1,051 |

**Supplementary Figure S1:** Doi plot of SBM Per week (CIC)


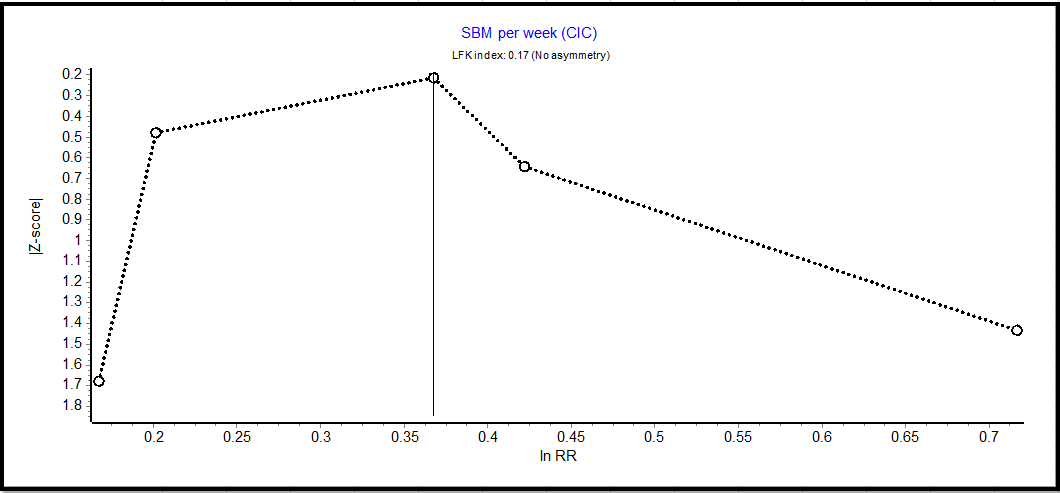


**Supplementary Figure S2:** Doi plot of SBM within 24 hours (CIC)


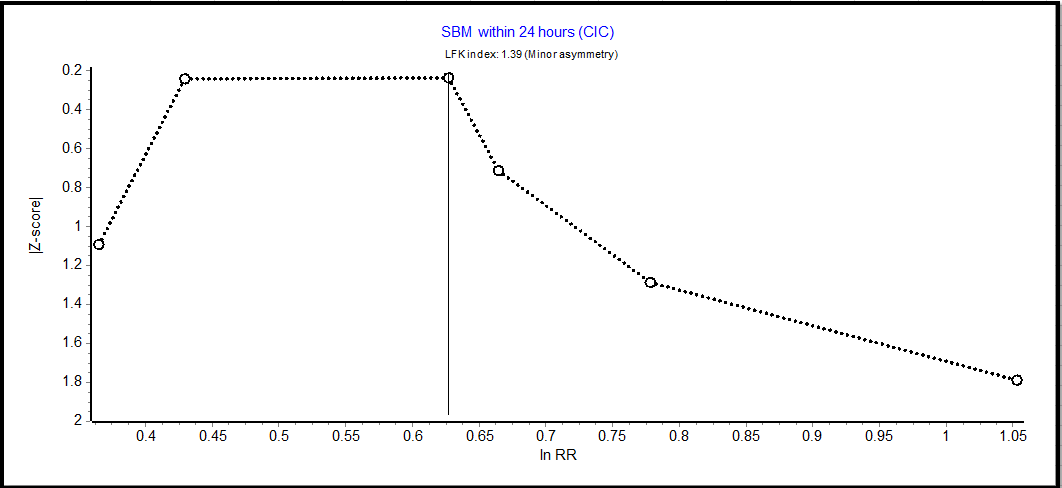


**Supplementary Figure S3:** Doi plot of Treatment Related Abdominal Pain (CIC)


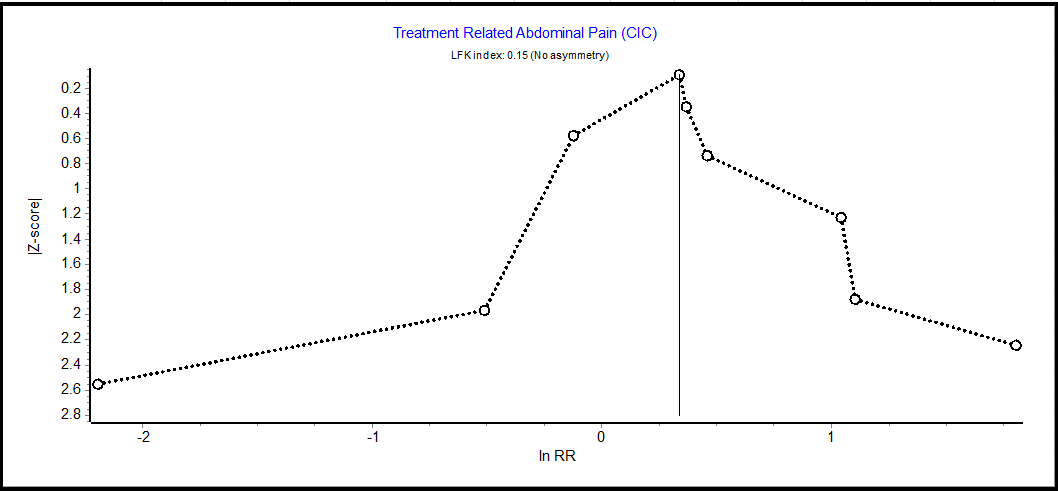


**Supplementary Figure S4:** Doi plot of SBM within 24 hours (OIC)


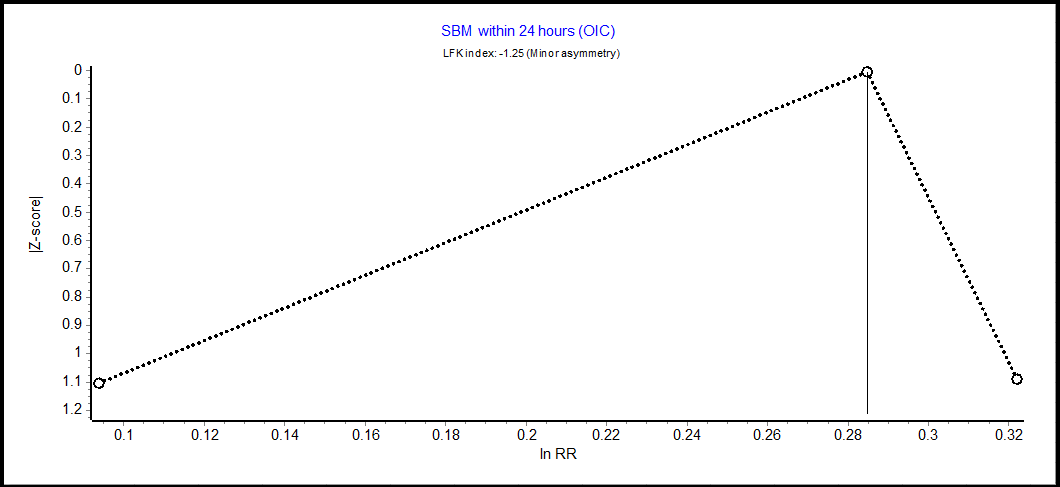


**Supplementary Figure S5:** Doi plot of Treatment Related Abdominal Pain (OIC)


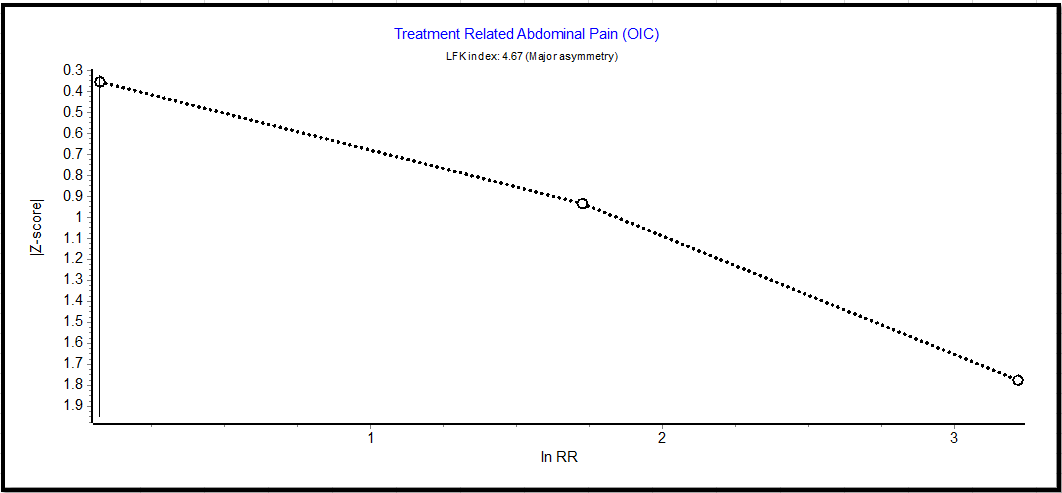

Supplement: Supplementary file 1 — Supplementary Figure S1: Doi plot of SBM Per week (CIC). Supplementary Figure S2: Doi plot of SBM within 24 h (CIC). Supplementary Figure S3: Doi plot of Treatment Related Abdominal Pain (CIC). Supplementary Figure S4: Doi plot of SBM within 24 h (OIC). Supplementary Figure S5: Doi plot of Treatment Related Abdominal Pain (OIC). Supplementary Table S1: Detailed Search Strategy of Each Database. [file JGH3-9-e70070-s001.docx]
